# Occupational role and COVID-19 among foreign-born healthcare workers in Sweden: a registry-based study

**DOI:** 10.1093/eurpub/ckad016

**Published:** 2023-02-10

**Authors:** Chioma Nwaru, Huiqi Li, Carl Bonander, Ailiana Santosa, Stefan Franzén, Maria Rosvall, Fredrik Nyberg

**Affiliations:** School of Public Health and Community Medicine, Institute of Medicine, Sahlgrenska Academy, University of Gothenburg, Gothenburg, Sweden; School of Public Health and Community Medicine, Institute of Medicine, Sahlgrenska Academy, University of Gothenburg, Gothenburg, Sweden; School of Public Health and Community Medicine, Institute of Medicine, Sahlgrenska Academy, University of Gothenburg, Gothenburg, Sweden; School of Public Health and Community Medicine, Institute of Medicine, Sahlgrenska Academy, University of Gothenburg, Gothenburg, Sweden; School of Public Health and Community Medicine, Institute of Medicine, Sahlgrenska Academy, University of Gothenburg, Gothenburg, Sweden; School of Public Health and Community Medicine, Institute of Medicine, Sahlgrenska Academy, University of Gothenburg, Gothenburg, Sweden; Social Medicine, Regionhälsan, Region Västra Götaland, Gothenburg, Sweden; School of Public Health and Community Medicine, Institute of Medicine, Sahlgrenska Academy, University of Gothenburg, Gothenburg, Sweden

## Abstract

**Background:**

Many studies report that foreign-born healthcare workers (HCWs) in high-income countries have an elevated risk of COVID-19. However, research has not yet specifically evaluated the distribution of COVID-19 among foreign-born workers in different healthcare work groups. We examined the risk of COVID-19 infection and hospitalization among foreign-born HCWs in different occupational roles in Sweden.

**Methods:**

We linked occupational data (2019) of 783 950 employed foreign-born workers (20–65 years) to COVID-19 data registered between 1 January 2020 and 30 September 2021. We used Cox proportional hazards regression to estimate the hazard ratio (HR) with 95% confidence intervals (95% CIs) of COVID-19 infection and hospitalization in eight healthcare occupational groups vs. non-HCWs and assessed whether region of birth modified the association between healthcare occupations and COVID-19.

**Results:**

All HCWs had a higher risk of COVID-19 outcomes than non-HCWs, but the risk differed by occupational role. Hospital-based assistant nurses had the highest risk (infection: HR 1.78; 95% CI 1.72–1.85; hospitalization: HR 1.79; 95% CI 1.52–2.11); allied HCWs had the lowest risk (infection: HR 1.22; 95% CI 1.10–1.35; hospitalization: HR 0.98; 95% CI 0.59–1.63). The relative hazard of the outcomes varied across foreign-born workers from different regions. For example, the relative risk of COVID-19 infection associated with being a physician compared to a non-HCW was 31% higher for African-born than European-born workers.

**Conclusions:**

The risk of COVID-19 among foreign-born HCWs differed by occupational role and immigrant background. Public health efforts that target occupational exposures as well as incorporate culturally responsive measures may help reduce COVID-19 risk among foreign-born HCWs.

## Introduction

The COVID-19 pandemic has tremendously impacted global health and economic well-being. As shown in studies from several western countries,^1–6^ both the risk and consequences of the pandemic are disproportionately higher in foreign-born populations or persons of Black, Asian and minority ethnic (BAME) groups compared to native-born populations or individuals of White ethnic backgrounds. In a systematic review published in 2021, Hayward *et al*.^6^ reported occupational exposure as one of the key determinants of the disproportionate risk of COVID-19 among the foreign-born population in high-income countries, a finding they linked to the overrepresentation of foreign-born populations in public-facing occupations known to be associated with increased risk of infection.

Healthcare is one such public-facing occupation. In Sweden, for example, where the foreign-born population represents 17% of the overall workforce, they constitute 26% of assistant nurses and 34% of practicing medical doctors according to national statistics in 2020.^7^ In the USA, the foreign-born population, although constituted 17% of the total workforce in 2018, represented 28% of physicians, 24% of dentists and 38% of home health aides.^8^ Of all occupational groups, healthcare workers (HCWs) have been the worst hit by COVID-19,^9–12^ and even in this sector, several reports suggest that foreign-born populations (compared to native-born individuals) and persons of non-White ethnic backgrounds (compared to individuals of white ethnic backgrounds) have been mostly affected.^10^^,^^13^^,^^14^ While some suggest^10^^,^^15^^,^^16^ that the overrepresentation of foreign-born HCWs in occupations (e.g. registered nurses, nursing assistants, home health aides) and clinical settings (e.g. in-patient hospitals, nursing homes or care homes) with greater exposure to SARS-CoV-2 might explain the increased risk overall in the broader group of foreign-born HCWs, epidemiological research has not yet specifically studied the distribution of COVID-19 in foreign-born HCWs in different occupational roles.

Current research suggests that certain ethnic groups (Blacks and Asians, e.g.) or foreign-born populations carry a greater risk of COVID-19 than others,^17^ but whether such a pattern also occurred among foreign-born HCWs remains unclear. Risk assessment is an important prerequisite to the effective management of COVID-19, and proper risk assessment requires an accurate understanding of individuals or staff groups most vulnerable to COVID-19.^18^ In this study, we aimed to investigate the distribution of COVID-19 infection and hospitalization among foreign-born HCWs in different occupational groups in Sweden and to assess whether the association between healthcare occupations and COVID-19 varied by region of birth.

## Methods

This nationwide, prospective observational study is based on register data from the SCIFI-PEARL (Swedish COVID-19 Investigation for Future Insights–a Population Epidemiology Approach using Register Linkage) project. The SCIFI-PEARL project was established in 2020 to provide timely and up-to-date responses to scientific questions regarding COVID-19 determinants and prognosis.^19^ The project includes health and administrative data from multiple databases and registers for the entire Swedish population. The current study is restricted to data of foreign-born individuals aged between 20 and 65 years (in 2020) who were alive and resident in Sweden on 1 January 2020 and employed based on the latest (2019) employment information obtained from the Longitudinal Integrated Database for Health Insurance and Labour Market Studies (LISA) (*N* = 953 334). We excluded individuals with missing information on occupation (*n* = 128 379), education level (*n* = 40 633) or with unknown country of birth information (*n* = 372), leaving 783 950 individuals. The Swedish Ethical Authority approved the study (2020-01800).

HCWs were classified based on their occupations in 2019. The information, registered as a four-digit Swedish Standard Occupational Classification (SSYK2012) code, was retrieved from the LISA register. All essential healthcare occupations as defined by Billingsley *et al*.^20^ were selected and used for exposure classification, plus a few additions informed by previous studies^9^ (Supplementary table S1). The healthcare occupations were grouped into eight categories: physicians, dentists, nurses, dental nurses/hygienists, hospital-based assistant nurses (including ambulance attendants), assistant nurses in elderly/homecare, home-based personal care workers (hereafter called personal care workers) and allied HCWs (physiotherapists, chiropractors, naprapaths, occupational therapist and health professionals not elsewhere classified). All workers in occupations except the selected healthcare occupations were defined as non-HCWs and used as the reference category in the analysis. Information on region of birth was obtained from the Total Population Register (TPR). The study participants were categorized into five geographical regions: Europe, Africa, Asia, USA/Canada/Oceania and Mexico/South/Central America/the Caribbean (hereafter called Latin America/Caribbean).

COVID-19 infection and COVID-19-related hospitalization were the outcomes of interest. An individual was defined as having a COVID-19 infection if he/she had a specialist healthcare encounter (visit or hospitalization) with an International Classification of Diseases 10th revision, Swedish version (ICD-10-SE) code U07.1 or U07.2 in the National Patient Register (NPR) or the same codes as an underlying or contributing cause of death in the Cause-of-Death Register, or a positive test result for SARS-CoV-2 in the national database of notifiable diseases (SmiNet). The event date was the earliest of these. An individual was defined as having a COVID-19-related hospitalization if he/she was admitted to the hospital based on a primary or secondary diagnosis of COVID-19. The event date was the date of hospital admission. Follow-up for each outcome started from 1 January 2020 to the earliest of the outcome, emigration, death or 30 September 2021.

Information on age (categorized as 20–34, 35–44, 45–54 and 55–65 years) and sex (men or women) were obtained from the TPR. From the LISA register, we obtained data on marital status (single, married/cohabiting or separated/divorced/widowed), education (primary, secondary or tertiary), annual gross individual income expressed in multiples of 100 SEK (<1000, 1000–2999, 3000–4999 and ≥5000) and county of residence, categorized as large or small depending on whether the county population was over a million inhabitants or not. From the NPR, we retrieved information based on ICD-10 SE codes for health conditions [hypertension, diabetes, stroke, obesity, asthma, chronic obstructive pulmonary disease, pneumonia and psychiatric conditions] diagnosed between 2015 and 2019 (Supplementary table S2). An individual with at least one of the listed health conditions was defined as having a comorbid condition. These variables were treated as potential confounders based on previous literature.^21–24^

Frequencies and percentages were used to describe study participants’ characteristics. Between-group comparisons were performed using Chi-square tests for categorical variables. Cox proportional hazard regression was used to estimate the risk of COVID-19 infection and hospitalization in foreign-born HCWs in the eight different occupational groups compared to non-HCWs. The analysis was performed in steps. Model I included age, sex, county of residence, region of birth and marital status. Model II additionally included education and income, and Model III additionally included comorbid conditions.

To assess whether region of birth modified the association between healthcare occupations and COVID-19 outcomes, Model III was reparameterized to include an interaction between occupation and region of birth. The log-likelihood ratio (LR) test was used to compare the models with and without the interaction term. Following the results, we fitted a stratified Cox regression using the *strata* option for *stcox* in Stata that included interactions between region of birth and all other variables in the model. The model is equivalent to running separate subgroup analyses by region of birth but enables formal testing of differences between regions of birth (here using Stata’s *lincom* command), i.e. the difference between regression coefficients of non-European-born (African-born, Asian-born, USA/Canada/Oceanian-born and Latin American/Caribbean-born) and European-born HCWs in the same occupations.

Since the risk of SARS-CoV-2 exposure may change in different waves of the pandemic, a sensitivity analysis was performed by restricting the data to only the first wave (1 January 2020–30 June 2020) to estimate the extent to which changes in SARS-CoV-2 exposure due to the pandemic intensity or character may have influenced the distribution of COVID-19 outcomes among foreign-born HCWs in different occupational groups. Because of the exclusion of individuals with missing information on some important variables (occupation, education and country of birth), an evaluation of possible selection bias was also conducted using the propensity score weighting method.^25^ To do this, we first calculated the probability of being included in the study using a logistic regression model, with baseline characteristics (age, sex, county of residence, marital status, income and comorbid condition) available for all individuals as predictors. Then we estimated the propensity scores for all included individuals, and finally reanalysed the data using weighted Cox proportional regression, with the inverse propensity score weights serving as the sample weight for the analyses.

The results were expressed as hazard ratios (HRs) and 95% confidence intervals (95% CIs). Statistical significance was established as *P* < 0.05. All statistical analyses were performed using Stata version 17 (StataCorp LLC, TX, USA).

## Results

We studied 783 950 employed foreign-born individuals, of whom 44% were born in Europe, 37% in Asia, 11% in Africa, 2% in USA/Canada/Oceania and 6% in Latin America or the Caribbean. The mean (SD) age of the study population was 42 (11.4) years and 51% were men. HCWs comprised 14% of the total population. Supplementary table S3 provides additional data on the distribution of baseline characteristics of the study population and the differences in the characteristics between HCWs and non-HCWs. HCWs were more likely to be women, divorced/widowed/separated and less likely to live in large cities than non-HCWs. The proportion of European-born workers was less among HCWs than among non-HCWs (37% vs. 46%). Among the HCWs, assistant nurses in elderly/homecare (40%), personal care workers (29%) and physicians (14%) were the largest occupational groups. In contrast, dentists (2%) and allied HCWs (2%) were few (table 1). European-born workers were overrepresented among physicians (61%), nurses (53%) and allied HCWs (64%), while in all the healthcare occupations, workers born in the USA/Canada/Oceania had 3% or less representation. Women were overrepresented in all the occupations, especially among dental nurses/hygienists (93%), nurses (87%), hospital-based assistant nurses (87%) and assistant nurses in homecare/elderly (85%) (table 1).

**Table 1:** Selected demographic characteristics of foreign-born individuals aged 20–65 years in different occupational groups within the healthcare sector in Sweden

	Occupational groups							
	Physicians	Nurses	Dentists	Dental nurses/hygienists	^a^Allied healthcare workers	Hospital-based assistant nurses	Assistant nurses in homecare/elderly	Personal care workers
	*N* = 14 041	*N* = 12 356	*N* = 2385	*N* = 3010	*N* = 1989	*N* = 9841	*N* = 39 913	*N* = 28 694
	*n* (%)	*n* (%)	*n* (%)	*n* (%)	*n* (%)	*n* (%)	*n* (%)	*n* (%)
**Age (years)**								
20–34	2685 (19.1)	2696 (21.8)	627 (26.3)	800 (26.6)	483 (24.3)	2902 (29.6)	8874 (22.2)	14 744 (51.4)
35–44	4803 (34.2)	3415 (27.6)	865 (36.3)	1009 (33.5)	551 (27.7)	2910 (28.6)	11 181 (28.0)	6567 (22.9)
45–54	3861 (27.5)	3504 (28.4)	528 (22.1)	749 (24.9)	464 (23.3)	2420 (24.6)	11 698 (29.3)	4433 (15.5)
55–65	2692 (19.2)	2741 (22.2)	365 (15.3)	452 (15.0)	491 (24.7)	1609 (16.3)	8160 (20.4)	2950 (10.3)
**Sex**								
Men	6780 (48.3)	1545 (12.5)	986 (41.3)	201 (6.7)	529 (26.6)	1261 (12.8)	6118 (15.3)	9553 (33.3)
Women	7261 (51.7)	10 811 (87.5)	1399 (58.7)	2809 (93.3)	1460 (73.4)	8580 (87.2)	33 795 (84.7)	19 141 (66.7)
**Region of birth**								
Europe	8591 (61.2)	6554 (53.0)	1029 (43.1)	1324 (44.0)	1281 (64.4)	3752 (38.1)	13 050 (32.7)	6223 (21.7)
Africa	493 (3.5)	1008 (8.2)	53 (2.2)	135 (4.5)	63 (3.2)	1531 (15.6)	9902 (24.8)	9065 (31.7)
Asia	4316 (30.7)	3739 (30.3)	1177 (49.4)	1351 (44.9)	450 (22.6)	3460 (35.2)	13 971 (35.0)	11 924 (41.6)
USA/Canada/Oceania	186 (1.4)	157 (1.3)	21 (0.9)	19 (0.6)	68 (3.4)	50 (0.5)	109 (0.3)	67 (0.2)
Latin America/Caribbean	445 (3.2)	898 (7.3)	105 (4.4)	181 (6.0)	127 (6.4)	1048 (10.7)	2881 (7.2)	1415 (4.9)

a Comprising chiropractors, naprapaths, physiotherapists, occupational therapists, and health professionals not elsewhere classified.

Between 1 January 2020 and 30 September 2021, we identified 141 938 cases of COVID-19 infection and 8138 cases of COVID-19-related hospitalization in the total sample. Eight percent of the identified COVID-19 infection cases and 30% of the COVID-19-related hospitalization cases occurred during the first wave of the pandemic, i.e. between 1 January 2020 and 30 June 2020. Table 2 shows the distribution of the COVID-19 cases in the different healthcare groups together with the HRs for the associations between healthcare occupations and COVID-19 outcomes. All healthcare work groups had increased risks of COVID-19 infection compared to non-HCWs in the unadjusted model. Adjustments for basic sociodemographic factors marginally influenced the estimates (Model I). Additional adjustment for socioeconomic characteristics increased the HRs for physicians, dentists, nurses and allied HCWs, and attenuated the estimates for hospital-based assistant nurses, assistant nurses in elderly/homecare and personal care workers (Model II). The estimates were essentially unaltered after additional adjustments for comorbid conditions (Model III). Hospital-based assistant nurses had the highest risk (HR 1.78; 95% CI 1.72–1.85), followed by nurses (HR 1.59; 95% CI 1.53–1.65) and assistant nurses in elderly/homecare (HR 1.55; 95% CI 1.52–1.58). Allied HCWs (HR 1.22; 95% CI 1.10–1.35) and dentists (HR 1.20; 95% CI 1.09–1.31) had the lowest risk (table 2).

**Table 2:** Associations between occupational groups in the healthcare sector and COVID-19-related infection and hospitalization among foreign-born workers aged 20–65 years in Sweden

	Number (%) of cases	Crude	Model I^a^	Model II^b^	Model III^c^
		HR (95% CI)	HR (95% CI)	HR (95% CI)	HR (95% CI)
**COVID-19 infection**					
Non-HCWs	115 280 (81.22)	1.00	1.00	1.00	1.00
Physicians	2738 (1.93)	1.20 (1.16–1.25)	1.19 (1.14–1.23)	1.36 (1.31–1.41)	1.36 (1.31–1.41)
Nurses	2874 (2.02)	1.47 (1.42–1.52)	1.48 (1.42–1.54)	1.59 (1.53–1.65)	1.59 (1.53–1.65)
Dentists	463 (0.33)	1.16 (1.06–1.27)	1.10 (1.00–1.20)	1.20 (1.09–1.31)	1.20 (1.09–1.31)
Dental nurses/hygienists	712 (0.50)	1.44 (1.34–1.55)	1.37 (1.27–1.48)	1.37 (1.28–1.48)	1.38 (1.28–1.48)
Allied healthcare workers^d^	367 (0.26)	1.11 (1.01–1.23)	1.15 (1.04–1.27)	1.22 (1.10–1.35)	1.22 (1.10–1.35)
Hospital-based assistant nurses	2854 (2.01)	1.91 (1.84–1.98)	1.90 (1.83–1.97)	1.79 (1.72–1.86)	1.78 (1.72–1.85)
Asst. nurses in elderly/homecare	10 232 (7.21)	1.63 (1.60–1.66)	1.66 (1.63–1.70)	1.55 (1.52–1.59)	1.55 (1.52–1.58)
Personal care workers	6418 (4.52)	1.37 (1.34–1.40)	1.41 (1.37–1.45)	1.38 (1.35–1.42)	1.38 (1.35–1.42)
Total population	141 938 (100)				
**COVID-19 hospitalization**					
Non-HCWs	6909 (84.90)	1.00	1.00	1.00	1.00
Physicians	157 (1.93)	1.09 (0.93–1.28)	1.05 (0.90–1.23)	1.41 (1.20–1.67)	1.40 (1.18–1.65)
Nurses	135 (1.66)	1.06 (0.90–1.26)	1.24 (1.05–1.47)	1.51 (1.26–1.79)	1.48 (1.24–1.76)
Dentists	34 (0.42)	1.39 (0.99–1.94)	1.37 (0.98–1.92)	1.69 (1.21–2.38)	1.67 (1.19–2.34)
Dental nurses/hygienists	31 (0.38)	1.00 (0.70–1.42)	1.26 (0.88–1.79)	1.30 (0.91–1.86)	1.33 (0.93–1.89)
Allied healthcare workers^d^	15 (0.18)	0.73 (0.44–1.22)	0.86 (0.52–1.42)	0.96 (0.58–1.60)	0.98 (0.59–1.63)
Hospital-based assistant nurses	152 (1.87)	1.50 (1.28–1.76)	1.86 (1.58–2.19)	1.85 (1.57–2.17)	1.79 (1.52–2.11)
Asst. nurses in elderly/homecare	448 (5.51)	1.09 (0.99–1.20)	1.18 (1.07–1.30)	1.15 (1.04–1.27)	1.13 (1.02–1.25)
Personal care workers	257 (3.16)	0.87 (0.77–0.98)	1.18 (1.04–1.34)	1.10 (0.97–1.25)	1.09 (0.96–1.24)
Total population	8138 (100)				

Hazards ratios (HRs) and 95% confidence intervals (95% CIs) were obtained from Cox proportional hazards regression models.

a Adjusted for age, sex, county of residence, region of birth and marital status.

b Adjusted for Model I, education and income.

c Adjusted for Model II and comorbid conditions.

d Comprising chiropractors, naprapaths, physiotherapists, occupational therapists and health professionals not elsewhere classified.

For COVID-19-related hospitalization (table 2), only the HR for hospital-based assistant nurses and personal care workers were statistically significant in the crude model. After adjusting for all potential confounders, five of the eight healthcare groups had statistically significant increased risks, with hospital-based assistant nurses being the group with the highest risk (HR 1.79; 95% CI 1.52–2.11). Dentists had 67% (HR 1.67; 95% CI 1.19–2.34) higher risk than non-HCWs, making them the group with the second-highest HR. For both outcomes, the HRs for non-hospital-based assistant nurses were lower than those of hospital-based assistant nurses.

Results from the LR tests showed a statistically significant interaction between occupation and region of birth both in the COVID-19 infection and COVID-19-related hospitalization models (*P* < 0.001 in each of the models). Table 3 presents a fully adjusted region of birth-stratified HRs for both outcomes. With the estimates, we computed the HRs comparing the risk of COVID-19 outcomes in European-born and non-European-born in the same healthcare occupation. The results (table 4) revealed some differences between the groups, especially in relation to COVID-19 infection. For example, the relative hazard of COVID-19 infection associated with being a physician or a dental nurse/hygienist compared to a non-HCW is higher for HCWs of African and Asian origin compared to HCWs of European origin. For COVID-19-related hospitalization, the HR associated with being a personal care worker compared to a non-HCW is 60% (HR 1.60, 95% CI 1.01–2.54) higher for Latin American/Caribbean-born workers compared to European-born workers.

**Table 3:** Region of birth-stratified associations between occupational groups in the healthcare sector and COVID-19-related infection and hospitalization observed among foreign-born workers aged 20–65 years in Sweden

	European-born	African-born	Asian-born	USA/Canada/Oceanian-born	Latin American/Caribbean-born
	^a^HR (95% CI)	^a^HR (95% CI)	^a^HR (95% CI)	^a^HR (95% CI)	^a^HR (95% CI)
**COVID-19 infection**					
Non-HCWs	1.00	1.00	1.00	1.00	1.00
Physicians	1.27 (1.21–1.35)	1.67 (1.38–2.02)	1.49 (1.40–1.59)	1.64 (1.18–2.28)	1.17 (0.93–1.47)
Nurses	1.56 (1.48–1.65)	1.72 (1.51–1.97)	1.57 (1.47–1.68)	1.99 (1.42–2.78)	1.73 (1.51–1.98)
Dentists	1.04 (0.89–1.21)	0.86 (0.41–1.81)	1.37 (1.22–1.54)	0.80 (0.20–3.20)	1.02 (0.64–1.63)
Dental nurses/hygienists	1.22 (1.08–1.38)	1.98 (1.44–2.72)	1.46 (1.31–1.62)	1.27 (0.41–3.96)	1.38 (1.02–1.86)
Allied healthcare workers^b^	1.24 (1.08–1.41)	0.85 (0.42–1.69)	1.22 (1.00–1.49)	0.92 (0.46–1.84)	1.59 (1.12–2.25)
Hospital-based assistant nurses	1.87 (1.76–1.99)	1.65 (1.49–1.84)	1.77 (1.67–1.88)	2.78 (1.67–4.64)	1.63 (1.45–1.84)
Asst. nurses in elderly/homecare	1.62 (1.56–1.68)	1.64 (1.56–1.72)	1.42 (1.37–1.47)	2.07 (1.39–3.08)	1.61 (1.49–1.74)
Personal care workers	1.34 (1.27–1.42)	1.47 (1.39–1.55)	1.34 (1.29–1.40)	2.00 (1.19–3.34)	1.48 (1.32–1.65)
**COVID-19 hospitalization**					
Non-HCWs	1.00	1.00	1.00	1.00	1.00
Physicians	1.25 (0.95–1.66)	2.00 (1.10–3.64)	1.44 (1.14–1.82)	2.79 (0.66–11.79)	1.80 (0.87–3.71)
Nurses	1.54 (1.16–2.05)	1.24 (0.69–2.23)	1.48 (1.14–1.93)	–	1.91 (1.13–3.24)
Dentists	1.83 (0.95–3.54)	–	1.71 (1.14–2.57)	–	1.09 (0.15–7.76)
Dental nurses/hygienists	0.71 (0.29–1.71)	2.49 (0.80–7.78)	1.72 (1.14–2.59)	–	–
Allied healthcare workers^b^	0.58 (0.21–1.54)	–	1.81 (0.97–3.38)	–	0.99 (0.14–7.05)
Hospital-based assistant nurses	2.20 (1.68–2.88)	1.78 (1.20–2.64)	1.49 (1.13–1.97)	–	1.73 (1.07–2.80)
Asst. nurses in elderly/homecare	1.07 (0.87–1.30)	1.03 (0.82–1.27)	1.15 (0.99–1.34)	1.47 (0.20–11.11)	1.26 (0.91–1.75)
Personal care workers	1.28 (0.98–1.68)	1.19 (0.93–1.51)	0.86 (0.70–1.05)	–	2.05 (1.41–2.99)

Hazards ratios (HRs) and 95% confidence intervals (95% CIs) were obtained from stratified Cox proportional hazards regression models.

a HR adjusted for age, sex, county of residence, marital status, education, income and comorbid conditions.

b Comprising chiropractors, naprapaths, physiotherapists, occupational therapists and health professionals not elsewhere classified.

**Table 4:** Region of birth heterogeneity in hazard ratios (HRs) for COVID-19 for healthcare workers (HCWs) vs. non-HCW among foreign-born individuals in Sweden aged 20–65 years

Region of birth		Occupational groups						
	Physicians	Nurses	Dentists	Dental nurses/hygienists	Allied healthcare workers^a^	Hospital-based assistant nurses	Assistant nurses in homecare/elderly	Personal care workers
	HR (95% CI)	HR (95% CI)	HR (95% CI)	HR (95% CI)	HR (95% CI)	HR (95% CI)	HR (95% CI)	HR (95% CI)
**COVID-19 infection**								
European-born	1.00	1.00	100	1.00	1.00	1.00	1.00	1.00
African-born	1.31 (1.08–1.59)	1.11 (0.96–1.27)	0.83 (0.39–1.78)	1.62 (1.15–2.28)	0.68 (0.34–1.39)	0.89 (0.78–1.00)	1.01 (0.95–1.07)	1.09 (1.01–1.18)
Asian-born	1.17 (1.07–1.27)	1.01 (0.93–1.10)	1.32 (1.09–1.61)	1.19 (1.02–1.40)	0.99 (0.78–1.25)	0.95 (0.87–1.03)	0.88 (0.83–0.92)	1.00 (0.93–1.07)
USA/Canada/Oceanian-born	1.29 (0.92–1.80)	1.28 (0.91–1.79)	0.77 (0.19–3.11)	1.04 (0.33–3.26)	0.74 (0.37–1.51)	1.49 (0.89–2.49)	1.28 (0.86–1.90)	1.48 (0.88–2.49)
Latin American/Caribbean-born	0.92 (0.73–1.16)	1.11 (0.96–1.28)	0.99 (0.61–1.61)	1.12 (0.81–1.56)	1.28 (0.88–1.86)	0.87 (0.76–1.00)	0.99 (0.91–1.08)	1.10 (0.97–1.24)
**COVID-19 hospitalization**								
European-born	1.00	1.00	100	1.00	1.00	1.00	1.00	1.00
African-born	1.60 (0.82–3.10)	0.81 (0.42–1.55)	–	3.52 (0.83–14.83)	–	0.81 (0.50–1.31)	0.96 (0.72–1.29)	0.93 (0.64–1.33)
Asian-born	1.15 (0.80–1.66)	0.96 (0.65–1.42)	0.93 (0.43–2.02)	2.42 (0.92–6.40)	3.14 (0.98–10.05)	0.68 (0.46–1.00)	1.08 (0.84–1.39)	0.67 (0.47–0.94)
USA/Canada/Oceanian-born	2.23 (0.51–9.68)	–	–	–	–	–	1.38 (0.18–10.53)	–
Latin American/Caribbean-born	1.44 (0.66–3.12)	1.24 (0.68–2.26)	0.59 (0.07–4.71)	–	1.71 (0.19–15.40)	0.79 (0.45–1.40)	1.18 (0.80–1.74)	1.60 (1.01–2.54)

The table shows ratios between estimated adjusted HRs among non-European-born vs. European-born HCWs and 95% confidence intervals (95% CIs) from a stratified Cox proportional hazards regression with interaction terms. The HRs, which were estimated using Stata’s *lincom* command, can be used to assess effect measure modification by region of birth. For example, the stratified HR for COVID-19 infection for African-born physicians vs. non-HCW is 1.67. The corresponding stratified HR for European-born physicians is 1.27. The HR for this comparison is 1.67/1.27 = 1.31, which implies that the relative hazard of COVID-19 infection associated with being a physician compared to non-HCWs was 31% higher for African-born compared to European-born workers. The stratified HRs can be found in Table 3.

a Comprising chiropractors, naprapaths, physiotherapists, occupational therapists and health professionals not elsewhere classified.

Restricting the data to only the first wave did not materially change the distribution of COVID-19 risk across the different healthcare groups, but did increase the magnitude of the risk estimates (Supplementary tables S4). We also detected region of birth differences in the risk of COVID-19 infection in the first wave (Supplementary tables S5 and S6) although the pattern slightly contrasted that observed when we analysed the entire study period. For example, in the first wave, the relative HR associated with being a physician or a personal care worker compared to a non-HCW was lower among HCWs of African and Asian descent compared to European-born in the same occupation, whereas the opposite was the case when we analysed the entire study period (1 January 2020–30 September 2021). The study findings were, however, very closely similar after accounting for possible selection bias using inverse propensity score weights calculated as a function of baseline characteristics (Supplementary tables S7–S9).

## Discussion

We analysed the risk of COVID-19 infection and COVID-19 hospitalization among foreign-born HCWs in different occupational groups using national Swedish population data. Our data revealed elevated risks of both outcomes among HCWs compared to non-HCWs, with the magnitude of the risk varying across occupational groups. The finding was similar when we restricted the analysis to only the first wave of the pandemic, although the magnitude of the risk estimates was larger, which is expected given the novelty of the infection and the lack of effective control measures during the early phase of the pandemic. We also observed that the relative risk of COVID-19 varied across foreign-born HCWs from different immigrant backgrounds.

A higher risk of COVID-19 infection and hospitalization overall among HCWs than non-HCWs has significant support in the existing literature.^10^^,^^12^ However, to our knowledge, this is the first population-representative longitudinal study to quantify the risk of these outcomes among foreign-born HCWs in different occupational groups, and the findings indicate different risks across different occupational groups. This finding is of public health importance, given that effective COVID-19 mitigation is dependent on accurate identification of the most vulnerable groups. Our finding regarding the disproportionate risk of COVID-19 among hospital-based assistant nurses corroborates several previous studies not specifically conducted among the foreign-born population group.^26–29^ Mandić-Rajčević *et al*.^29^ hypothesized that the disproportionate risk among assistant nurses could be due to their frequent and close proximity to patients and patients’ bodily fluids since they are more often than other HCWs responsible for caring for the hygiene, transport, and manoeuvring of sick patients. Interestingly, the HRs for the outcomes among non-hospital-based assistant nurses (i.e. those working in homecare and elderly care homes) were lower than that of hospital-based assistant nurses. In a previous Swedish study on seroprevalence among HCWs, Ocias *et al*.^30^ found an increased prevalence of seropositivity among assistant nurses in hospital-based healthcare, but not in assistant nurses working in elderly care homes. These findings point to the crucial role the work environment plays in the transmission of the SARS-CoV-2 and support the recommendation by Khunti *et al*.^18^ that the workplace, workforce, and individual factors all be considered when making risk assessments for COVID-19 prevention and control among the working population.

We found that dentists had the second largest HRs for COVID-19-related hospitalization. Exposure to the SARS-CoV-2 virus is expected to be high among dentists because of the nature of their work, which typically involves working in close contact with colleagues and patients in a closed environment, and the use of aerosol-generating procedures.^31^ However, evidence from previous studies^31^^,^^32^ suggests that the increased exposure among dentists can successfully be mitigated by strict adherence to COVID-19 infection prevention and control measures, such as frequent screening of patients and staff members for COVID-19, disinfecting between patients and encouraging social distancing between patients. As we do not have data on COVID-19 precautionary measures among foreign-born dentists, it is challenging to estimate the extent to which better or worse adherence to COVID-19 prevention and control measures influenced our findings. While previous studies^33^^,^^34^ reported high adherence to COVID-19 recommendations among the foreign-born population in general, it remains unclear how different foreign-born HCWs responded to workplace COVID-19 prevention guidelines. Considering that dentists in this study had a lower HR for COVID-19 infection and a higher HR for COVID-19-related hospitalization than most other HCWs (also seen when the data were restricted to only the first wave), it is possible that they may be reasonably well-protected against infection, but if they get infected, the dose may be higher which could increase severity and hospitalization. Evidence of an association between high viral load and COVID-19 disease severity have been reported in previous studies, although the findings are not clearcut.^35^ The results regarding dentists could also be due to chance finding resulting from multiple testing. There is a need for more studies on the pattern of COVID-19 outcomes among foreign-born dentists in Sweden.

Our finding of differential risk of COVID-19 across regions of birth in some healthcare occupations aligns with studies from the UK^5^^,^^36^ and the USA^37^ that showed ethnic variations in the risk of COVID-19 among HCWs. This finding may be explained by group differences in genetics, culture, religion and lifestyle characteristics, which are likely to influence perceptions of risk and attitudes towards COVID-19-related prevention and control measures.^33^^,^^38^ Future studies may consider how culture and lifestyle characteristics impact the adherence to COVID-19 prevention measures of foreign-born HCWs from different immigrant backgrounds, as that would inform better tailoring of COVID-19 mitigation strategies.

This study has some limitations. First, our sample is focused on first-generation immigrants as it does not include people born to immigrant parents residing in Sweden since they are defined as Swedish-born in the Swedish Population Register. Second, since we used employment data registered in 2019, there is a risk that some individuals’ occupational status may have changed during the study, although we do not expect a substantial change given the great need for HCWs during the pandemic. Third, there might be a problem of residual confounding due to the lack of data on living arrangements (e.g. household size) and work characteristics such as the work setting (hospital or non-hospital), workplace locality (COVID-19 ward or not), contract type (permanent or temporary) and employment type (full-time or not). Finally, even though we used data from all foreign-born workers in Sweden within the studied age range, we could not estimate the risk of COVID-19-related hospitalization in some groups because of a small number of observations. Sample size also limited our ability to explore in greater details potential differences within some immigrant groups (e.g. sub-Saharan vs. North African HCWs). On the other hand, using high-quality register data alleviates some potential problems relating to differential misclassification of exposure and recall bias. The prospective design in a population-based setting, with a relatively long follow-up, and large sample size are additional strengths of the study. With the rich data available, we were also able to control for a range of important potential confounders. Our findings are generalizable to foreign-born HCWs (aged 20–65 years) in Sweden.

In conclusion, the current study showed a disparity in the risk of COVID-19 infection and COVID-19-related hospitalization across foreign-born HCWs in different occupational groups. For both COVID-19 outcomes, hospital-based assistant nurses had the most elevated risk of all the healthcare groups. The relative risk of the outcomes also varied by region of birth. These findings suggest that multiple factors put foreign-born HCWs at risk of COVID-19. Public health efforts should not only target work-related exposures but also incorporate culturally responsive measures to ensure effective prevention and control of COVID-19 in this population group and across these occupations.

## Supplementary Material

ckad016_Supplementary_Data

## Data Availability

The data underlying this article cannot be shared publicly due to their containing sensitive information that could compromise the privacy of research participants. The data will be shared upon reasonable request to the corresponding author.
