# Supplementary material for: Occupational role and COVID-19 among foreign-born healthcare workers in Sweden: a registry-based study
Source: Eur J Public Health. 2023 Feb 10;33(2):202–8. doi: 10.1093/eurpub/ckad016 (PMC10066486; doi:10.1093/eurpub/ckad016)
Supplement: ckad016_Supplementary_Data [file ckad016_supplementary_data.docx]

Occupational variation in the risk of Covid-19 infection and hospitalization among foreign-born healthcare workers in Sweden: a registry-based study

Supplementary Table S1: Essential healthcare occupations and codes based on the Swedish Standard for Classification of Occupations (SSYK 2012)

| **SSYK12** | **Occupations** |
| --- | --- |
| 2211 | Specialist physicians |
| 2212 | Resident physicians |
| 2213 | General practitioners |
| 2219 | Other physicians |
| 2260 | Dentist |
| 2221 | Professional nurses |
| 2222^a^ | Professional midwives |
| 2223 | Anesthesia nurses |
| 2224 | District nurses |
| 2226 | Nurses- ambulance |
| 2227 | Nurses-geriatric |
| 2228 | Nurses- intensive care |
| 2231 | Nurses-operation |
| 2232 | Nurses-children |
| 2235 | Nurses-radiology |
| 2239 | Other specialist nurses |
| 2289 | Health professionals not elsewhere classified |
| 2271 | Chiropractors and naprapaths |
| 2272 | Physiotherapists |
| 2273 | Occupational therapist |
| 2289 | Health professionals not elsewhere classified |
| 3250^a^ | Dental hygienists |
| 5350 | Dental nurses |
| 5321 | Assistant nurses, homecare, and homes for the elderly |
| 5322 | Assistant nurses, rehabilitation |
| 5323 | Assistant nurses, hospital ward |
| 5324 | Assistant nurses, clinic |
| 5325 | Assistant nurses, children |
| 5326 | Ambulance attendants |
| 5330 | Home-based personal care and related workers |

The selection of the occupations was based on the work of Billingsley et al.^1^

^a^ The author’s own additions based on previous research.^2^

**Reference:**

1. Billingsley S, Brandén M, Aradhya S, Drefahl S, Andersson G, Mussino E. Deaths in the frontline: Occupation-specific COVID-19 mortality risks in Sweden. Stockholm Reports in Demography 2020. <https://doi.org/10.17045/sthlmuni.12816065.v2>.
2. Mutambudzi M, Niedwiedz C, Macdonald EB, et al. Occupation and risk of severe COVID-19: a prospective cohort study of 120 075 UK Biobank participants. Occup Environ Med 2021;78:307-314.

Supplementary Table S2. Comorbid conditions and their International Classification of Disease codes

| **Comorbid conditions** | **ICD codes (version 10)** |
| --- | --- |
| Hypertension | 110-115 |
| Stroke | 160, 161, 162, 1630-1635, 1638-1639, 164 |
| Psychiatric conditions | F20 F21, F22, F23, F24, F25, F26, F27, F28, F29, F30, F31, F32, F33, F34, F35, F36, F37, F38, F39 |
| Diabetes | E10, E11 |
| Pneumonia | J10-J18 |
| COPD^a^ | J44 |
| Asthma | J45 |
| Obesity | E66 |

^a^ Chronic obstructive pulmonary disease

Supplementary Table S3. Baseline characteristics of employed foreign-born individuals aged 20-65 years in Sweden, overall and subdivided into healthcare and non-healthcare workers

|  | Total sample  N = 783,950 | Healthcare workers  n = 112,229 | Non-healthcare workers  n = 671,721 | P-value |
| --- | --- | --- | --- | --- |
| Age (years)  20-34  35-44  45-54  55-65 | 234,765 (30.0)  231,284 (29.5)  185,220 (23.6)  132,681 (16.9) | 30.1  27.9  24.6  17.3 | 29.9  29.8  23.5  16.9 | <0.001 |
| Sex  Men  Women | 398,237 (50.8)  385,713 (49.2) | 24.0  76.0 | 55.3  44.7 | <0.001 |
| Size of the county of residence  Small (< 1 million population)  Large (> 1 million population) | 276,824 (35.3)  507,126 (64.7) | 42.6  57.4 | 34.1  65.9 | <0.001 |
| Region of birth  Africa  Asia  Europe  USA/Canada/Oceania  Latin America/Caribbean | 87,919 (11.2)  287,558 (36.7)  348,155 (44.4)  12,598 (1.6)  47,720 (6.1) | 19.8  36.0  37.3  0.6  6.3 | 9.8  36.8  45.6  1.8  6.1 | <0.001 |
| Marital status  Single  Married/cohabiting  Divorced/widowed/separated | 234,283 (29.9)  412,836 (52.7)  136,831 (17.5) | 27.5  49.8  22.7 | 30.3  53.1  16.6 | <0.001 |
| Education  Primary  Secondary  Tertiary | 112,725 (14.4)  300,872 (38.4)  370,353 (47.2) | 8.8  44.8  46.4 | 15.3  37.3  47.4 | <0.001 |
| Individual annual gross income  <1000 SEK  1000-2999 SEK  3000-4999 SEK  ≥5000 SEK | 72,568 (9.3)  260,093 (33.2)  337,590 (43.1)  113,699 (14.5) | 8.2  36.8  39.4  15.5 | 9.4  32.6  43.7  14.3 | <0.001 |
| Comorbid condition  No  Yes | 762,719 (97.3)  21,231 (2.7) | 87.2  12.8 | 89.1  10.9 | <0.001 |

Supplementary Table S4: Associations between occupational groups in the healthcare sector and Covid-19-related infection and hospitalisation reported during the first wave (1 January 2020 – 30 June 2020) of the pandemic among foreign-born workers aged 20-65 years in Sweden. Hazards ratios (HR) and 95% confidence intervals (95%CI) were obtained from Cox proportional hazards regression models

|  | Number (%) of cases | Crude  HR (95%CI) | Model I†  HR (95%CI) | Model II‡  HR (95%CI) | Model III§  HR (95%CI) |
| --- | --- | --- | --- | --- | --- |
| **Covid-19 infection** |  |  |  |  |  |
| Non-HCWs  Physicians  Nurses  Dentists  Dental nurses/hygienists  Allied healthcare workers*  Hospital-based assistant nurses  Asst. nurses in elderly/homecare  Personal care workers  Total population | 6692 (57.42)  526 (4.51)  628 (5.39)  43 (0.37)  54 (0.46)  62 (0.53)  664 (5.70)  2033 (17.44)  953 (8.18)  11655 (100) | 1.00  3.83 (3.51-4.19)  5.24 (4.82-5.69)  1.81 (1.34-2.45)  1.80 (1.38-2.35)  3.17 (2.47-4.07)  7.00 (6.46-7.58)  5.23 (4.98-5.49)  3.37 (3.15-3.61) | 1.00  3.87 (3.54-4.23)  5.15 (4.74-5.60)  1.75 (1.30-2.36)  1.73 (1.32-2.26)  3.35 (2.61-4.31)  6.72 (6.20-7.30)  4.77 (4.53-5.04)  3.32 (3.10-3.57) | 1.00  4.51 (4.09-4.98)  5.76 (5.28-6.29)  1.98 (1.47-2.68)  1.78 (1.36-2.33)  3.75 (2.92-4.83)  6.25 (5.75-6.79)  4.42 (4.19-4.68)  3.24 (3.02-3.48) | 1.00  4.48 (4.06-4.95)  5.71 (5.23-6.23)  1.97 (1.46-2.66)  1.79 (1.37-2.34)  3.77 (2.93-4.85)  6.17 (5.68-6.71)  4.39 (4.16-4.64)  3.23 (3.01-3.47) |
| **Covid-19 hospitalisation** |  |  |  |  |  |
| Non-HCWs  Physicians  Nurses  Dentists  Dental nurses/hygienists  Allied healthcare workers*  Hospital-based assistant nurses  Asst. nurses in elderly/homecare  Personal care workers  Total population | 1961 (79.59)  59 (2.39)  59 (2.39)  13 (0.53)  10 (0.41)  5 (0.20)  70 (2.84)  188 (7.63)  99 (4.02)  2464 (100) | 1.00  1.44 (1.11-1.87)  1.64 (1.26-2.12)  1.87 (1.08-3.22)  1.14 (0.61-2.12)  0.86 (0.36-2.07)  2.44 (1.92-3.09)  1.61 (1.39-1.87)  1.18 (0.97-1.45) | 1.00  1.46 (1.12-1.89)  1.93 (1.49-2.51)  1.91 (1.10-3.29)  1.47 (0.79-2.75)  1.04 (0.43-2.51)  2.93 (2.30-3.74)  1.65 (1.41-1.93)  1.49 (1.21-1.83) | 1.00  1.98 (1.51-2.62)  2.44 (1.86-3.19)  2.44 (1.41-4.22)  1.58 (0.85-2.95)  1.23 (0.51-2.98)  2.98 (2.33-3.81)  1.65 (1.41-1.94)  1.38 (1.12-1.70) | 1.00  1.97 (1.49-2.59)  2.38 (1.82-3.12)  2.40 (1.38-4.15)  1.62 (0.87-3.02)  1.26 (0.52-3.03)  2.89 (2.26-3.69)  1.62 (1.38-1.90)  1.37 (1.11-1.68) |

† Adjusted for age, sex, county of residence, region of birth, and marital status

‡ Adjusted for Model I, education, and income

§ Adjusted for Model II and comorbid conditions

*Comprising chiropractors, naprapaths, physiotherapists, and occupational therapists

Supplementary Table S5**:** Region of birth-stratified associations between occupational groups in the healthcare sector and Covid-19-related infection and hospitalisation reported during the first wave (1 January 2020 – 30 June 2020) of the pandemic among foreign-born workers aged 20-65 years in Sweden. Hazards ratios (HR) and 95% confidence intervals (95%CI) were obtained from stratified Cox proportional hazards regression models.

|  | European-born | African-born | Asian-born | USA/Canada/Oceanian-born | Latin American/Caribbean-born |
| --- | --- | --- | --- | --- | --- |
|  | †HR (95%CI) | †HR (95%CI) | †HR (95%CI) | †HR (95%CI) | †HR (95%CI) |
| **Covid-19 infection** |  |  |  |  |  |
| Non-HCWs  Physicians  Nurses  Dentists  Dental nurses/hygienists  Allied healthcare workers*  Hospital-based assistant nurses  Asst. nurses in elderly/homecare  Personal care workers | 1.00  4.63 (4.01-5.35)  6.75 (5.92-7.69)  2.32 (1.41-3.79)  1.32 (0.76-2.28)  4.32 (3.10-6.03)  7.44 (6.45-8.58)  5.20 (4.70-5.75)  4.02 (3.46-4.67) | 1.00  3.36 (2.11-5.37)  5.03 (3.77-6.70)  1.62 (0.23-11.54)  0.60 (0.08-4.27)  2.79 (0.69-11.19)  4.58 (3.67-5.72)  3.37 (2.97-3.84)  2.94 (2.55-3.39) | 1.00  4.35 (3.72-5.07)  4.84 (4.17-5.61)  1.90 (1.28-2.82)  2.14 (1.53-3.00)  3.05 (1.86-4.99)  5.92 (5.19-6.74)  4.23 (3.89-4.60)  3.05 (2.74-3.39) | 1.00  12.96 (5.88-28.59)  19.77 (9.71-40.23)  ------  ------  3.70 (0.51-27.23)  20.29 (7.76-53.04)  8.94 (3.32-24.08)  17.17 (6.52-45.19) | 1.00  4.56 (2.76-7.53)  5.81 (4.27-7.92)  0.96 (0.13-6.87)  2.31 (0.95-5.59)  5.73 (2.70-12.16)  5.48 (4.24-7.08)  4.50 (3.72-5.44)  2.50 (1.83-3.42) |
| **Covid-19 hospitalisation** |  |  |  |  |  |
| Non-HCWs  Physicians  Nurses  Dentists  Dental nurses/hygienists  Allied healthcare workers*  Hospital-based assistant nurses  Asst. nurses in elderly/homecare  Personal care workers | 1.00  1.51 (0.91-2.50)  3.11 (2.01-4.82)  3.42 (1.27-9.22)  1.32 (0.33-5.33)  0.64 (0.09-4.55)  4.54 (3.00-6.87)  1.84 (1.31-2.58)  2.28 (1.49-3.50) | 1.00  2.12 (0.83-5.38)  2.35 (1.12-4.91)  ------  2.30 (0.32-16.53)  ------  1.86 (1.00-3.45)  1.00 (0.71-1.41)  1.36 (0.95-1.95) | 1.00  2.24 (1.54-3.26)  2.39 (1.60-3.58)  2.41 (1.24-4.68)  1.87 (0.88-3.94)  1.87 (0.63-6.13)  2.69 (1.81-4.01)  1.87 (1.48-2.37)  0.84 (0.57-1.25) | 1.00  11.00 (2.29-52.88)  ------  ------  ------  ------  ------  ------  ------ | 1.00  2.02 (0.48-8.55)  0.93 (0.23-3.81)  ------  ------  3.10 (0.43-22.39)  2.24 (1.08-4.65)  1.86 (1.14-3.06)  2.71 (1.55-4.74) |

†HR adjusted for age, sex, county of residence, marital status, education, income, and comorbid conditions

*Comprising chiropractors, naprapaths, physiotherapists, and occupational therapists

Supplementary Table S6: Region of birth heterogeneity in hazard ratios (HRs) for Covid-19 for healthcare workers (HCWs) versus non-HCW among foreign-born individuals in Sweden aged 20-65 years. The table shows ratios between estimated adjusted HRs among non-European-born versus European-born HCWs and 95% confidence intervals (95%CI) from a stratified Cox proportional hazards regression with interaction terms. The HR ratios, which were estimated using Stata’s *lincom* command, can be used to assess effect measure modification by region of birth. For example, the stratified HR for Covid-19 infection for African-born physicians versus non-HCW is 3.36. The corresponding stratified HR for European-born physicians is 4.63. The HR ratio for this comparison is 3.36/4.63 = 0.73, which implies that the relative hazard of Covid-19 infection associated with being a physician compared to non-HCWs was lower for African-born compared to European-born workers. The stratified HRs can be found in Supplementary Table S5

| Region of birth |  | Occupational groups | | | | | | |
| --- | --- | --- | --- | --- | --- | --- | --- | --- |
|  | Physicians | Nurses | Dentists | Dental nurses/hygienists | Allied healthcare workers* | Hospital-based assistant nurses | Assistant nurses in elderly/homecare | Personal care workers |
|  | HR ratio (95%CI) | HR ratio (95%CI) | HR ratio  (95%CI) | HR ratio  (95%CI) | HR ratio  (95%CI) | HR ratio (95%CI) | HR ratio (95%CI) | HR ratio (95%CI) |
| **Covid-19 infection** |  |  |  |  |  |  |  |  |
| European-born  African-born  Asian-born  USA/Canada/Oceanian-born  Latin America/Caribbean-born | 1.00  0.73 (0.45-1.19)  0.94 (0.76-1.16)  2.80 (1.25-6.26)  0.99 (0.58-1.66) | 1.00  0.75 (0.54-1.02)  0.72 (0.59-0.87)  2.93 (1.42-6.03)  0.86 (0.62-1.20) | 1.00  0.70 (0.09-5.30)  0.82 (0.43-1.54)  --------  0.42 (0.05-3.15) | 1.00  0.45 (0.06-3.49)  1.63 (0.86-3.09)  --------  1.75 (0.62-4.95) | 1.00  0.64 (0.15-2.69)  0.71 (0.39-1.28)  0.86 (0.11-6.48)  1.33 (0.58-3.02) | 1.00  0.62 (0.47-0.80)  0.80 (0.66-0.97)  2.73 (1.03-7.21)  0.74 (0.55-0.99) | 1.00  0.65 (0.55-0.76)  0.81 (0.71-0.93)  1.72 (0.64-4.66)  0.87 (0.70-1.07) | 1.00  0.73 (0.59-0.90)  0.76 (0.63-0.91)  4.27 (1.60-11.38)  0.62 (0.44-0.88) |
| **Covid-19 hospitalisation** |  |  |  |  |  |  |  |  |
| European-born  African-born  Asian-born  USA/Canada/Oceanian-born  Latin America/Caribbean-born | 1.00  1.41 (0.49-4.07)  1.49 (0.79-2.80)  7.31 (1.40-38.05)  1.34 (0.29-6.19) | 1.00  0.75 (0.32-178)  0.77 (0.42-1.39)  --------  0.30 (0.07-1.31) | 1.00  --------  0.70 (0.21-2.32)  --------  -------- | 1.00  1.75 (0.16-19.44)  1.41 (0.29-6.85)  --------  -------- | 1.00  --------  3.08 (0.32-29.86)  --------  4.86 (0.30-78.96) | 1.00  0.41 (0.19-0.86)  0.59 (0.33-1.05)  --------  0.49 (0.21-1.14) | 1.00  0.56 (0.44-0.88)  1.02 (0.67-1.54)  --------  1.02 (0.56-1.85) | 1.00  0.60 (0.34-1.04)  0.37 (0.21-0.66)  --------  1.19 (0.59-2.40) |

*Comprising chiropractors, naprapaths, physiotherapists, and occupational therapists

Supplementary Table S7: Associations between occupational groups in the healthcare sector and Covid-19 among foreign-born workers aged 20-65 years in Sweden. Hazards ratios (HR) and 95% confidence intervals (95%CI) were obtained from weighted Cox proportional hazards regression models.

|  | HR (95%CI) ^a^ |
| --- | --- |
| **Covid-19 infection** |  |
| Non-HCWs  Physicians  Dentists  Nurses  Hospital-based assistant nurses  Asst. nurses in elderly/homecare  Personal care workers  Dental nurses/hygienists  Allied healthcare workers^b^ | 1.00  1.36 (1.30-1.41)  1.20 (1.09-1.32)  1.58 (1.52-1.65)  1.79 (1.72-1.86)  1.54 (1.51-1.58)  1.40 (1.36-1.43)  1.38 (1.28-1.49)  1.20 (1.08-1.34) |
| **Covid-19 hospitalization** |  |
| Non-HCWs  Physicians  Dentists  Nurses  Hospital-based assistant nurses  Asst. nurses in elderly/homecare  Personal care workers  Dental nurses/hygienists  Allied healthcare workers^b^ | 1.00  1.40 (1.18-1.65)  1.68 (1.19-2.36)  1.48 (1.24-1.76)  1.77 (1.50-2.08)  1.13 (1.02-1.25)  1.06 (0.93-1.21)  1.32 (0.92-1.88)  0.98 (0.58-1.66) |

^a^ HR adjusted for age, sex, county of residence, region of birth, marital status, education, income, and comorbid conditions

^b^ Comprising chiropractors, naprapaths, physiotherapists, and occupational therapists

Supplementary Table S8: Region of birth-stratified associations between occupational groups in the healthcare sector and Covid-19 outcomes among foreign-born workers aged 20-65 years in Sweden. Hazards ratios (HR) and 95% confidence intervals (95%CI) were obtained from weighted stratified Cox proportional hazards regression models.

|  | European-born | African-born | Asian-born | USA/Canada/Oceanian  -born | Latin America/Caribbean-born |
| --- | --- | --- | --- | --- | --- |
|  | HR (95%CI) ^a^ | HR (95%CI) ^a^ | HR (95%CI) ^a^ | HR (95%CI) ^a^ | HR (95%CI) ^a^ |
| **Covid-19 infection** |  |  |  |  |  |
| Non-HCWs  Physicians  Dentists  Nurses  Hospital-based assistant nurses  Asst. nurses in elderly/homecare  Personal care workers  Dental nurses/hygienists  Allied healthcare workers^b^ | 1.00  1.27 (1.20-1.34)  1.04 (0.89-1.22)  1.55 (1.47-1.64)  1.87 (1.76-2.00)  1.62 (1.55-1.68)  1.35 (1.28-1.43)  1.23 (1.08-1.39)  1.20 (1.05-1.38) | 1.00  1.66 (1.37-2.02)  0.85 (0.40-1.78)  1.74 (1.52-1.99)  1.68 (1.50-1.88)  1.64 (1.56-1.72)  1.48 (1.40-1.56)  1.97 (1.43-2.73)  0.80 (0.39-1.61) | 1.00  1.48 (1.39-1.58)  1.37 (1.22-1.55)  1.57 (1.47-1.68)  1.78 (1.67-1.89)  1.42 (1.37-1.47)  1.36 (1.30-1.41)  1.48 (1.33-1.64)  1.23 (1.00-1.51) | 1.00  1.67 (1.18-2.35)  0.75 (0.19-3.03)  1.96 (1.38-2.78)  2.73 (1.60-4.67)  2.07 (1.37-3.13)  2.06 (1.20-3.51)  1.17 (0.39-3.55)  0.95 (0.47-1.91) | 1.00  1.18 (0.93-1.49)  1.03 (0.65-1.63)  1.73 (1.51-1.99)  1.66 (1.46-1.88)  1.60 (1.47-1.73)  1.51 (1.35-1.69)  1.37 (1.01-1.85)  1.66 (1.16-2.39) |
| **Covid-19 hospitalization** |  |  |  |  |  |
| Non-HCWs  Physicians  Dentists  Nurses  Hospital-based assistant nurses  Asst. nurses in elderly/homecare  Personal care workers  Dental nurses/hygienists  Allied healthcare workers^b^ | 1.00  1.25 (0.94-1.65)  1.77 (0.92-3.43)  1.56 (1.16-2.08)  2.19 (1.67-2.87)  1.07 (0.87-1.30)  1.30 (0.98-1.71)  0.70 (0.29-1.70)  0.54 (0.20-1.45) | 1.00  1.98 (1.10-3.57)  ------  1.26 (0.70-2.29)  1.69 (1.14-2.51)  1.00 (0.80-1.24)  1.14 (0.90-1.46)  2.40 (0.77-7.50)  ------ | 1.00  1.45 (1.14-1.83)  1.75 (1.16-2.64)  1.49 (1.14-1.94)  1.47 (1.11-1.95)  1.16 (0.99-1.35)  0.82 (0.67-1.01)  1.70 (1.12-2.58)  1.91 (0.99-3.66) | 1.00  2.84 (0.68-11.88)  ------  ------  ------  1.43 (0.19-10.96)  ------  -----  ------ | 1.00  1.75 (0.86-3.56)  1.02 (0.15-7.15)  1.86 (1.10-3.16)  1.72 (1.06-2.80)  1.26 (0.90-1.77)  2.06 (1.41-3.03)  ------  0.88 (0.12-6.27) |

^a^ HR adjusted for age, sex, county of residence, marital status, education, income, and comorbid conditions

^b^ Comprising chiropractors, naprapaths, physiotherapists, and occupational therapists

Supplementary Table S9: Region of birth heterogeneity in hazard ratios (HRs) for Covid-19 for healthcare workers (HCW) versus non-HCW among foreign-born individuals in Sweden aged 20-65 years. The table shows ratios between estimated adjusted HRs among non-European-born versus European-born HCW and 95% confidence intervals (95%CI) from a stratified Cox proportional hazards regression with interaction terms. The HR ratios, which were estimated using Stata’s *lincom* command, can be used to assess effect measure modification by region of birth. For example, the stratified HR for Covid-19 infection for African-born physicians versus non-HCW is 1.66. The corresponding stratified HR for European-born physicians is 1.27. The HR ratio for this comparison is 1.66/1.27 = 1.31, which implies that the relative hazard of Covid-19 infection associated with being a physician compared to a non-HCW was 31% higher for African-born compared to European-born workers. The stratified HRs can be found in Supplementary Table S8

| Region of birth |  | Occupational groups | | | | | | |
| --- | --- | --- | --- | --- | --- | --- | --- | --- |
|  | Physicians | Dentists | Nurses | Hospital-based assistant nurses | Asst. nurses in elderly/homecare | Personal care workers | Dental nurses/hygienists | Allied healthcare workers ^a^ |
|  | HR ratio  (95%CI) | HR ratio  (95%CI) | HR ratio  (95%CI) | HR ratio  (95%CI) | HR ratio  (95%CI) | HR ratio (95%CI) | HR ratio  (95%CI) | HR ratio  (95%CI) |
| **Covid-19 infection** |  |  |  |  |  |  |  |  |
| European-born  African-born  Asian-born  USA/Canada/Oceanian  -born  Latin America/Caribbean-born | 1.00  1.31 (1.07-1.60)  1.17 (1.07-1.27)  1.31 (0.93-1.86)  0.93 (0.73-1.18) | 1.00  0.81 (0.38-1.74)  1.32 (1.08-1.61)  0.73 (0.18-2.95)  0.99 (0.61-1.61) | 1.00  1.12 (0.97-1.30)  1.01 (0.93-1.11)  1.26 (0.88-1.80)  1.11 (0.96-1.30) | 1.00  0.90 (0.79-1.02)  0.95 (0.87-1.04)  1.46 (0.85-2.50)  0.89 (0.77-1.02) | 1.00  1.01 (0.95-1.08)  0.88 (0.83-0.93)  1.28 (0.84-1.94)  0.99 (0.90-1.08) | 1.00  1.09 (1.01-1.18)  1.00 (0.94-1.08)  1.52 (0.89-2.61)  1.11 (0.98-1.27) | 1.00  1.61 (1.14-2.28)  1.21 (1.03-1.42)  0.96 (0.31-2.91)  1.12 (0.81-1.55) | 1.00  0.66 (0.32-1.35)  1.02 (0.80-1.31)  0.79 (0.39-1.61)  1.38 (0.94-2.04) |
| **Covid-19 hospitalization** |  |  |  |  |  |  |  |  |
| European-born  African-born  Asian-born  USA/Canada/Oceanian  -born  Latin America/Caribbean-born | 1.00  1.58 (0.83-3.04)  1.16 (0.81-1.67)  2.27 (0.53-9.78)  1.40 (0.65-3.01) | 1.00  --------  0.99 (0.45-2.14)  --------  0.57 (0.07-4.49) | 1.00  0.81 (0.42-1.57)  0.96 (0.64-1.42)  --------  1.20 (0.66-2.19) | 1.00  0.77 (0.48-1.25)  0.67 (0.46-1.00)  --------  0.79 (0.45-1.38) | 1.00  0.93 (0.69-1.26)  1.09 (0.84-1.40)  1.34 (0.17-10.38)  1.18 (0.80-1.75) | 1.00  0.88 (0.61-1.27)  0.63 (0.45-0.90)  --------  1.59 (0.99-2.56) | 1.00  3.42 (0.81-14.42)  2.42 (0.91-6.40)  --------  -------- | 1.00  --------  3.51 (1.09-11.42)  --------  1.62 (0.18-14.55) |

^a^ Comprising chiropractors, naprapaths, physiotherapists, and occupational therapists
